# Climate Response and Radial Growth Dynamics of Pedunculate Oak (*Quercus robur* L.) Plus Trees and Their Half-Sib Progeny in Periods of Severe Droughts in the Forest-Steppe Zone of Eastern Europe

**DOI:** 10.3390/plants13223213

**Published:** 2024-11-15

**Authors:** Daria A. Litovchenko, Anna A. Popova, Konstantin A. Shestibratov, Konstantin V. Krutovsky

**Affiliations:** 1 Department of Forestry, Forest Taxation and Forest Management, G.F. Morozov Voronezh State University of Forestry and Technologies, 394087 Voronezh, Russia; timashchuk90@mail.ru; 2 Department of Forest Genetics, Biotechnology and Plant Physiology, G.F. Morozov Voronezh State University of Forestry and Technologies, Timiryazeva Str. 8, 394087 Voronezh, Russia; logachevaaa@rambler.ru; 3 Shemyakin-Ovchinnikov Institute of Bioorganic Chemistry, Russian Academy of Sciences, 6 Prospect Nauki, 142290 Pushchino, Russia; 4 Department of Forest Genetics and Forest Tree Breeding, Georg-August University of Göttingen, 37077 Göttingen, Germany; 5 Center for Integrated Breeding Research, George-August University of Göttingen, 37075 Göttingen, Germany; 6 Laboratory of Forest Genomics, Genome Research and Education Center, Institute of Fundamental Biology and Biotechnology, Siberian Federal University, 660041 Krasnoyarsk, Russia; 7 Department of Genomics and Bioinformatics, Institute of Fundamental Biology and Biotechnology, Siberian Federal University, 660041 Krasnoyarsk, Russia; 8 Laboratory of Population Genetics, N. I. Vavilov Institute of General Genetics, Russian Academy of Sciences, 119333 Moscow, Russia; 9 Scientific and Methodological Center, G. F. Morozov Voronezh State University of Forestry and Technologies, 394087 Voronezh, Russia

**Keywords:** dendrochronology, dendrophenotype, radial growth, pedunculate oak, plus tree, drought, forest-steppe zone

## Abstract

The dendrochronological parameters of 97 pedunculate oak (*Quercus robur* L.) trees including 20 plus trees (142-year-old on average) and four half-sib families for four of them were analyzed considering also specifically years of the most severe droughts that were identified using average monthly air temperature and precipitation data. The tree-ring width (TRW) was mostly affected by air temperature that had the largest cross-dating indices (CDI), up to 78% maximum. However, the 32-year Brückner–Egeson–Lockyer cycle (a climatic cycle of approximately 30–40 years that correlates with sunspot activity) was more reflected in the TRW dynamics in plus trees than precipitation and air temperature. A high-frequency of abnormal TRW was clearly observed during drought periods and in the following 2–3 years. Tree radial-growth reduction due to drought stress varied significantly between families. The resistance to drought based on TRW was higher in the maternal plus oak trees than in progeny. Drought resulted in reduced growth during the subsequent year(s); hence, the minimum growth occurred after the actual climate event. Autumn–winter precipitation and weather conditions were of the greatest importance at the onset of active vegetation in April and May. The influence of air temperature on oak growth was the largest in March (*r* = 0.39, *p* < 0.05). The strongest positive correlation between precipitation and growth (with *r* up to 0.38) was observed in May 2023. Plus trees had a high adaptive potential due to the stability of radial growth during drought with high resistance (Rt = 1.29) and resilience (Rs = 1.09) indexes. The offspring of families 1 (Rt = 0.89, Rs = 0.89) and 2 (Rt = 1.04, Rs = 0.87) had similar resistance and resilience, but the recovery indices (Rc) for offspring in families 1, 2 and 3 exceeded the recovery values for plus trees. For offspring in families 3 and 4, the index values were lower. The revealed responses of wood growth of plus trees to climatic parameters estimated as resistance (Rt), resilience (Rs) and recovery (Rc) indexes and similar responses in their progeny can be used in breeding pedunculate oak for wood growth productivity and drought resistance.

## 1. Introduction

Current global climate warming has a particularly strong impact on the growth, condition and survival of woody plants [1,2,3,4,5]. Existing climate models show that forest ecosystems may become endangered due to an increase in the frequency and intensity of droughts [6,7,8]. Herewith, it is questionable whether the natural plasticity and adaptive potential of forest tree species will be sufficient to survive and maintain the existing forest tree habitats [9,10,11,12,13]. Dendrochronological methods are an effective tool to study the response of woody plants to current and past changes in climatic factors, enabling its identification at different time periods [14,15,16,17]. The range of methods for dendroclimatic analysis is expanding; studies are reported that combine dendrochronological phenotyping and genotyping of trees [18,19,20,21,22]. Dendrochronological phenotyping makes it possible to manage more effectively forest ecosystems and predict their condition under the influence of climatic factors. For forest-forming and economically important tree species, in order to preserve them, numerous studies have been carried out to investigate their response, the genetic basis of sustainability, growth and productivity [23,24,25,26,27] and ecosystem management [28,29,30,31,32]. The most vulnerable forest ecosystems are fragmented forests at the edge of the habitat range [33]. Pedunculate oak (*Quercus robur* L.) is an economically significant species in Europe and Central Russia [34,35]. The southern border of its distribution is limited by the forest-steppe; in steppe regions, oak is confined to river floodplains. Special genetic reserves of this species have been established to protect it in such vulnerable areas, such as the Shipov Forest in Central Russia on the territory of the East European Plain. For the excellent quality of timber wood, Peter the Great declared this forest area the sovereign’s ship timber in 1709. In 1875, forester N.K. Genko took measures to restore Shipov Forest. Subsequently, such forestry masters as D.M. Kravchinsky, G.F. Morozov and N.K. Genko conducted research there. Nowadays, Shipov Forest is a stronghold for research work on pedunculate oak; oak plus trees are most often found there.

Most dendrochronological studies of pedunculate oak are devoted to predicting the growth of oak forests in response to climate change towards warming and increasing intensity and duration of droughts. The data based on tree rings and collected so far indicate irregularity of weather anomalies over time [36,37]. However, a reliable and thorough analysis of changes in the frequency of weather extremes over time is possible only by supplementing the series of instrumental meteorological with indirect data. This goal is achieved with the help of historical and climatological studies of chronicles and other documents [38,39] in combination with studying the dynamics of tree rings affected by anomalous climate events [40]. The results of such studies showed both the presence of a pronounced climate response in pedunculate oak and dependence of TRW on populations and their locations. Dendrochronological data for pedunculate oak have been obtained in many regions of western and central Europe. A significant variability of climate response depending on many factors, including the origin and geographic location of populations, has been shown [41,42,43]. In most of the temperate climate zones of Europe and Asia, the radial growth of woody plants has a positive correlation with the amount of precipitation in the spring–summer period. For instance, pedunculate oak in southern Sweden has a close positive correlation between radial growth and precipitation in June and July [44]. Precipitation from March through May was the primary limiting factor for intra-annual tree-ring growth of *Quercus robur* L. in southern Romania [45]. Pedunculate oak in the Czech Republic demonstrated a positive correlation of radial growth with precipitation in May, June and July [46,47]. In Estonia, regional growth patterns were similar to each other, but spatial differences in the response of growth to climate change were evident in the allocation of leading climate factors for each region (summer precipitation and temperature, particularly, June temperature and July precipitation) [48]. In the floodplain oak forests of the Tellerman Forestry (Voronezh Region, Russia), the radial growth of pedunculate oak closely correlated with precipitation in the same growing season [49]. Patlai and Gaida [50] noted the variability in growth of oak trees of different geographical origin with an amplitude of 42% already in three-year-old geographical cultures of the second generation of oak in the Ukrainian forest-steppe (type of forest growth conditions D2), with oak trees from the Central forest-steppe having the best characteristics. Despite the numerous dendrochronological studies on oaks in the Zagros Mountains [51,52,53], the effects of specific local site and stand conditions associated with different land-use types on the climate–growth relationships of *Quercus* have not been investigated thus far. Moreover, different, or even contrasting, findings concerning the dominant climatic drivers of *Quercus* growth, i.e., responses to precipitation [54] and air temperature [55], were found in Central Zagros. Degen et al. [56] noted in their study that the climatic response in radial growth can be determined by genetic and/or environmental factors—microheterogeneity of habitats, which can enhance or weaken the influence of air temperature and precipitation at the level of individual trees. Despite the variety of issues being considered, practical oak breeding remains detached from the study of biodiversity at all levels, the population structure of oak forests, and the use of the results of many years of testing of progeny populations. Defoliation by insects, infection by phytopathogens, reduced rainfall, increased temperature and longer dry seasons are some of the most significant causes of oak decline [57,58,59,60,61]. The differences in TRW stability in pedunculate oak, depending on the health of the trees, show that healthy trees maintain growth and have a high adaptive potential even under drought conditions. At the same time, drought resistance and TRW in weakened trees decreased sharply [62,63]. The survival of oak trees in arid conditions is highly dependent on the trees’ ability to adapt structurally, physiologically and genetically. Drought tolerance in oaks is adaptive [64], and changes in drought-related traits either evolve genetically [65] or arise through environmentally determined plasticity [66]. In this regard, it is necessary to accumulate data that will allow us to predict tree reactions more accurately, taking into account their genotypes and habitats, and to facilitate the development of selection models based on the characteristics of dendrophenotypes.

For pedunculate oak, special attention is paid to phenological features of leaf budding timing (phenoforms). Phenology offers critical insights into the responses of species to climate change [67]. There are several studies of the long-term dynamics of growth and phenology of leaves, the xylogenesis of two phenoforms of oak (early and late phenological varieties of leaf budding timing) and their sensitivity to meteorological factors [68,69,70]. The available comparative studies of the long-term dynamics of xylogenesis of the two phenological forms and their sensitivity to meteorological factors [68,69,70,71,72] show the absence of a homogeneous climatic response, and the presence of individual patterns of leaf and xylem development, which indicates a large genetic variability [69]. Along with phenoforms, plus trees are singled out. These are trees that are significantly superior in one trait or a set of economically valuable traits and properties to surrounding trees of the same age that grow in the same conditions. In even-aged, pure, high-density forest stands, to be considered as plus trees, they have to significantly exceed the average tree stand for the corresponding phenological form, for example, in height by 10% or more, in diameter by 30% or more [73]. Furthermore, plus trees in a population are clearly distinguished by strong apical dominance, longevity, high crown position, and straight trunk, and are, thus, part of intraspecific diversity. The dendrochronological characteristics, climate response and its intrapopulation variations, and the possibility of inheriting the dendrophenotypic characteristics of pedunculate oak plus trees have been poorly studied.

Based on the silvicultural and biological differences of plus trees and the uniqueness of the Shipova Forest population among large oak ecosystems, we assume that both general features of the climate response of plus trees and individual dendrophenotypic reactions are present in this population.

We hypothesized that plus trees and their offspring have a high adaptive potential to withstand climatic stresses such as droughts. Therefore, the main objective of this study was to analyze indices of important adaptive dendrophenotypes (resistance, resilience and recovery), intraspecific variability and response of individual pedunculate oak plus trees and their families to climatic factors in the forest-steppe zone of Central Russia.

## 2. Results

### 2.1. Climate Analysis

According to the Voronezh Meteorological Station, the average annual values of total (*T*av), minimum (*T*min) and maximum (*T*max) air temperatures, and total average precipitation (*P*_total_av) in the studied region were, according to the World Meteorological Organization (WMO) base period of 30-year averaging of climate indicators, as follows: for 1991–2020: *T*av = 6.1 °C ± 0.003, *T*min = 1.9 °C ± 0.001, *T*max = 10.6 °C ± 0.01, and *P*_total_av = 580 mm, and for 1987–2016: *T*av = 7.2 °C ± 0.002, *T*min = 2.9 °C ± 0.001, *T*max = 11.5 °C ± 0.01, and *P*_total_av = 603 mm. Since 2007, the average annual temperature has not fallen below 8.7 °C; a record low value of 7 °C was registered in 2015.

The amount of precipitation in the research region over the entire observation period (1879–2022) at the Voronezh Meteorological Station varied significantly from 263 mm in 1891 to 874 mm in 2012. The unevenness of precipitation was characteristic of the warm period.

The amount of precipitation for April–September was 182 mm in 2009, but 556 mm for this period in 2012 (anomalous 64% of the total annual amount of 874 mm in 2012), and again only 227 mm in 2014. Over twenty years (1990–2010), the average annual air temperature increased by 0.4 °C compared to the climate norm, mainly due to an increase in winter temperatures. The average annual precipitation decreased slightly by 4 mm. Climate continentality decreased from 49.1% (1937–1966) to 44.2% (1967–1996) [74,75]. Climate change, which has continued to this day, is characterized by a sharp increase in temperature (especially in cold seasons), a decrease in precipitation in the warm season, and, consequently, more frequent droughts.

**Figure 1:**
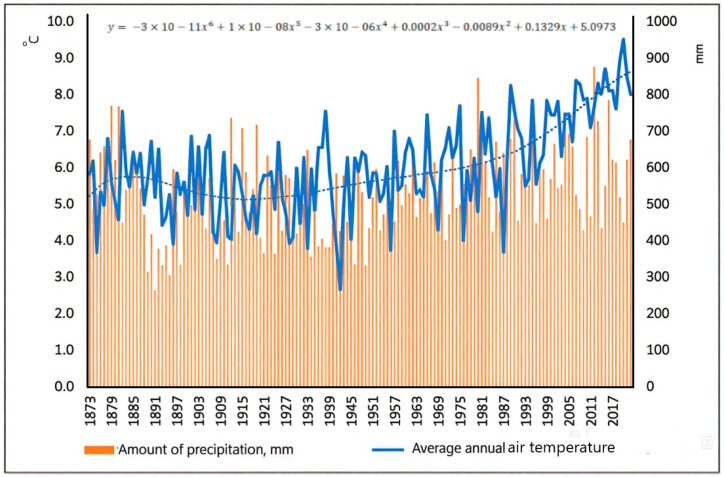
Total precipitation and average annual air temperature registered for the studied region by the Voronezh Meteorological Station.

Thus, the analysis of climatic data of the research region showed (1) a continuous intensive increase in average annual air temperature, mainly due to increased winter temperatures, and (2) a slight increase in average annual precipitation against the background of its cyclical fluctuations (Figure 1).

The total amount of precipitation in the summer of 2010 was 682 mm, above average for the periods 1991–2021 and 1987–2016. However, the drought in the summer of 2010 was critical for the radial growth of pedunculate oak. This mainly affected the radial growth of 2011. The drought of 2010 mainly occurred in the second half of the vegetation period, as evidenced by the annual-ring late-wood width, which ranged from 0.5 to 1.9 mm for late wood.

Matveev et al. [74] showed that the climatic situation in the summer of 2010, and especially on 29 July (the date of the fire in Usmansky Bor), was unique in a number of parameters (air temperature, wind conditions), but was not unusual in terms of precipitation.

Having analyzed the amount of precipitation and the average July temperature in the dry years that have been identified by us, we observed that precipitation in July 2010 was 54% of the norm, which is not a too low value, but temperatures in July and throughout the warm period this year were abnormally high. According to the Voronezh Meteorological Station data, a maximum air temperature of 38 °C was registered in June, July and August 2010. Such high temperature was observed before only in July 1938 and August 1946. The average monthly temperature in July 2010 reached 26.4 °C (Table 1). The previous maximum (24.9 °C) was observed in July 1938.

In years of severe droughts, the amount of precipitation in July decreased to 30–40 and even 10–15 mm. The years of the most severe droughts, accompanied by catastrophic fires (1939, 1972, 2010) and characterized by a deficit of precipitation for at least two previous years and extremely high average and maximum air temperatures of the warm period (May–August), were the most critical for tree ring growth.

### 2.2. Statistical and Relative Indicators of Oak Radial Growth Chronologies

The results of statistical analysis of radial growth are presented in Table 2. The radial growth of tree samples differed statistically significantly between all studied chronologies according to Student’s *t*-test (*p* ≤ 0.01).

The coefficient of mean sensitivity (MS) of chronology ranged from 0.25 to 0.29, which corresponds to the average sensitivity level of oak growth in the study area (the chronology is considered sensitive, if the MS is more than 0.3).

The calculated expressed population signal (EPS) value was 0.99 on average for the entire study period, i.e., the chronology can be considered sufficiently representative in accordance with the accepted threshold value of 0.85; that is, the TRW for each year adequately reflects the growth of the entire population.

The calculated signal-to-noise ratio (SNR) was 78, i.e., the generalized chronology contains high variability explained by the influence of climatic factors (that is, the trees under study were sensitive to changes in climatic conditions).

**Figure 2:**
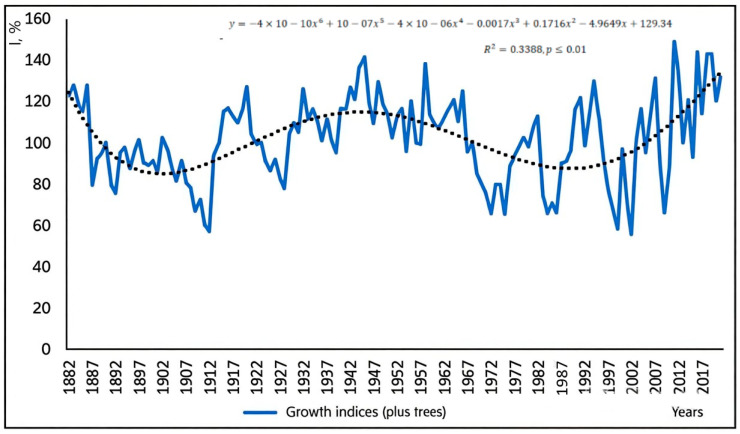
Average annual growth indices for 30 plus trees.

The diagram of radial growth indices of plus oak trees clearly shows years with an abnormally fast growth (1944–1945, 1958, 1990, 1998, 2004, 2007, 2011) and with an abnormally slow growth (1912, 1928, 1972, 1975, 1982, 1999, 2002, 2009). However, there are years when growth was very variable and in different trees varied from very slow to very fast in 1930, 1943, 1949, 1954, 1969, 1981, 1991, 1995, 2003, 2013 years, as reflected by the average values in the chart in Figure 2.

**Figure 3:**
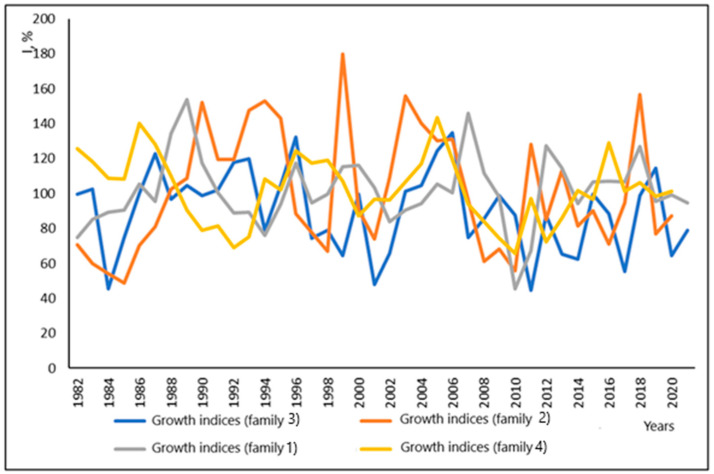
Average annual growth indices for trees in four families (1–4).

Average annual growth indices of trees in four families (Figure 3) show high fluctuation across different years, but similarity between families. Analysis showed a significant impact of droughts on the TRW in the studied families, but not always proportional to the severity of the drought. The strongest impact of drought was in 2010–2011 with deep minima of the growth indices. The highest amplitude of fluctuations was observed for family 2 (Figure 3).

The most significant drop in growth was noted in 2001, 2011 and 2017 in family 3, 1985, 1998, 2010 and 2016 in family 2, and 2010 in family 1. The fluctuations in radial growth in family 4 were rather smooth.

The highest growth was noted in 1999, 2003 and 2018 in family 2, 1989 and 2007 in family 1; in other families it was not clearly pronounced.

The main reason for the reduced growth observed in many trees during the drought years was the weather conditions in the previous years. The formation of narrow growth rings in most of the investigated trees occurred in the years characterized by a combination of several climatic anomalies: late spring frosts and dry summers.

It was found that the formation of anomalous rings occurred after extremely high temperatures of the warm period (May–August). The main key climatic factors affecting the annual growth of plus oak trees were, first, air temperature for the calendar year, and then, the amount of precipitation for the calendar year. The same was found for Siberian larch (*Larix sibirica* Ledeb.) growing on the south slopes of the Altay Mountains, especially in recent decades [76].

Air temperature as an indicator had the greatest effect, 78%. Since the obtained values of the Fisher criterion for all factors were greater than the standard (critical) threshold value (*Ff* (28.9) > *Fst* (3.9)), it can be concluded that the influence of climatic factors, such as air temperature and precipitation, on the oak growth were significant.

### 2.3. Analysis of the Coefficients of Similarity of Tree-Ring Width (TRW) Chronologies

Analysis of the synchronicity coefficient (GLK) values for individual tree-ring chronologies with an average chronology showed that the range of fluctuations among families was large, 40–90% (Table 3). A relatively low synchrony of changes in the magnitude of radial growth in some samples (GLK ≤ 67%) indicates differences of trees within these samples. The maximum levels of differentiation and variability (GLK = 79–100%) were observed in the years with an optimal combination of environmental factors. The maximum coefficient of variation of growth in trees for all families and plus trees was noted in years of droughts, as well as in two years with the highest level of spring-summer precipitation (1990 and 2012), which shows an individual response to climate within each family and plus trees.

Regarding the GLK of individual chronologies of the oak tree growth in the studied families, different levels of synchronicity were observed according to [77] or different levels of general convergence of a series of curves according to [78]. ARW values of 66%, 60% and 62% in families 1, 3 and 4, respectively, correspond to a low level, 78% in family 2—to an average level, and 81% in plus trees—to a high level according to [77].

The GSL for almost all families was “1–3” except several samples in which there was no synchronicity (“0”). In all samples collected from plus trees the average level of synchronicity reached “3”.

The CC (%) was even more variable than the GLK. The average CC for ARW for plus trees was 79%, which is considered as a significant level. Samples No. 8 and No. 14 in the family 2 showed values of only 41% and 45% for ARW, respectively, i.e., a moderate correlation with the average. However, for early and late wood, these samples showed a higher correlation. Sample No. 8 showed a CC value of 65% for late wood (a significant correlation), and 70% for early wood (a stronger correlation).

The CDI values were the largest for plus trees, ranging from 78% for ARW to 80% for LWW. The lowest values of the CDI (10%) and GLK (31%) were observed in the tree No. 2 in the family 4 for early wood.

### 2.4. Analysis of the Cyclicity of Radial Growth Fluctuations

**Figure 4:**
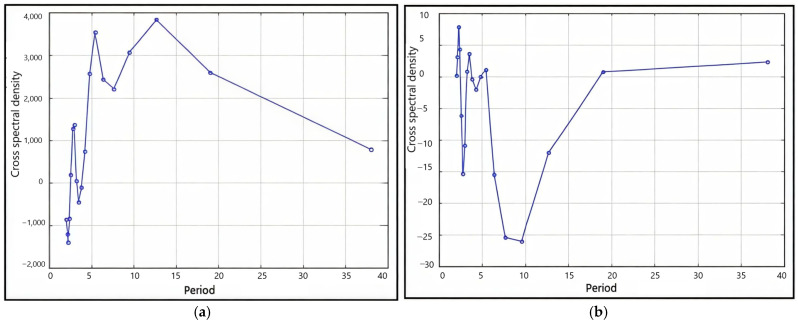
Periodogram of the cross-spectral density of the progeny series in four families by the period, smoothed using Hamming weights: (**a**) total annual atmospheric precipitation (*P*_year_), (**b**) air temperature (*t*).

To associate the radial growth of oak trees in plus trees and four families with climatic factors, such as total annual precipitation and air temperature, we carried out a cross-spectral analysis of the progeny radial growth series based on the total radial growth in four families (Figure 4).

It showed that the total TRW and atmospheric precipitation correlated following 12-, 6-, 9-, 4-, and 18-year-long periods. The 4–6-year-long periods were the most pronounced. When analyzing the cross-spectral density of air temperature series and growth indices for ARW, 2–3-year-long periods were the most pronounced; the prevalence of an 18-year-long period was also revealed (Figure 4b).

**Figure 5:**
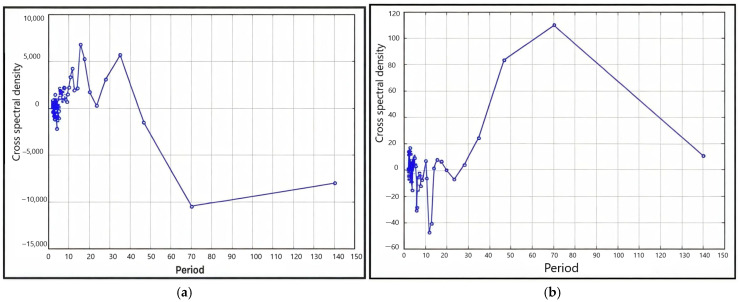
Periodogram of the cross-spectral density of the series of plus trees smoothed using Hamming weights: (**a**) total annual atmospheric precipitation (*P*_year_), (**b**) air temperature (*t*).

Cross-spectral analysis of the growth and precipitation indices revealed the Brückner–Egeson–Lockyer cycle (32-year-long periods) and the 12-year cycle to be pronounced (Figure 5). Analysis of the cross-spectral density of the series of the growth and air-temperature indices revealed that the 70-year-long periods were the most pronounced; the prevalence of the 46-year cycle was also revealed (Figure 5b).

The identified radial growth dynamics for early and late wood (a long-term trend and the 12- and 2–5-year cycles) and their correlations were typical, manifested in each tree studied.

### 2.5. Identification of Climate Response in the Radial Growth of Pedunculate Oak

We analyzed correlations between the climatic factors and different chronologies and examined the differences in resistance (*Rt*), resilience (*Rs*) and recovery (*Rc*) indexes between families and plus trees in response to drought stress (2010–2012 years) (Table 4).

**Figure 6:**
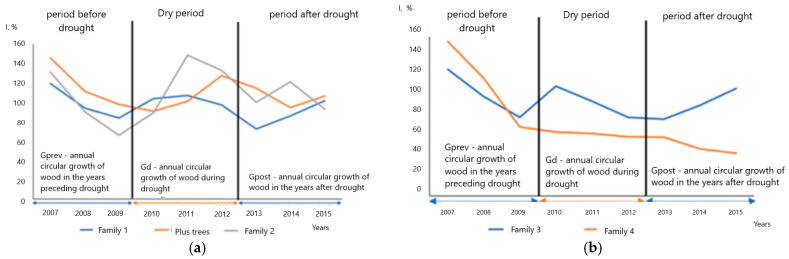
Average growth indices (I, %) before, during and after drought (2010–2012 years) in families 1 and 2, and plus trees (**a**), and in families 3 and 4 (**b**).

The results showed that the reduction in tree radial growth due to drought stress varied significantly between families (Figure 6), indicating differences in physiological and ecological regulation strategies. The resistance of plus oak trees to drought was higher than that of the families.

**Figure 7:**
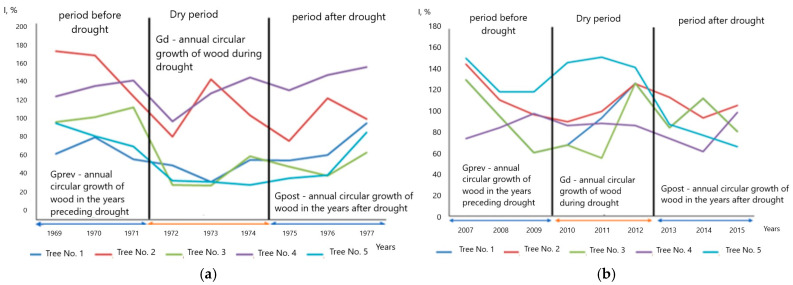
Examples of growth indices (I, %) for individual plus trees with pronounced climatic response before, during and after drought: (**a**) drought of 1972, (**b**) drought of 2010.

Analysis of plus trees by the indices presented above showed that the difference in growth in individual plus trees was less significant than in the families, which can be due to a greater sensitivity of younger trees in the families to environmental factors, namely, drought (Figure 7).

In general, indexes reflect the droughts, but sometimes with a delay. For instance, in the plus tree No. 5, drought in 2010 caused a slower growth in the following years, and the minimal growth occurred after the actual climate event. However, in some trees, indexes did not reflect the droughts, such as in the plus tree No. 2, whose growth did not reflect droughts, neither in 1972, nor in 2010, contradicting the assumption of decreased growth during the droughts.

Analysis of the radial growth indices for the previous 3 years before the droughts in 1972 and 2010 revealed a more stable growth of plus trees in comparison with their progeny in the families 1–4, and it continued over the next 3 years after the droughts with small differences. A small surge in growth activity was noted in some trees in the second year after the abnormally low growth in 2011 following the drought in 2010. A consistently low growth was recorded in 2010 in most trees. For this year, the highest average monthly temperatures during the growing season were observed (26.4 °C in July, 25.4 °C in August, 22.5 °C in June). In addition, very high temperatures were also registered in the summer months in the subsequent years 2011 and 2016 (up to 23.7 °C in July 2011), which could have a negative impact on the pattern of annual growth. However, the annual growth indices in individual trees are affected not only by various climatic factors, but also by their genotypes, position in the forest stand, soils, etc. [79,80].

**Figure 8:**
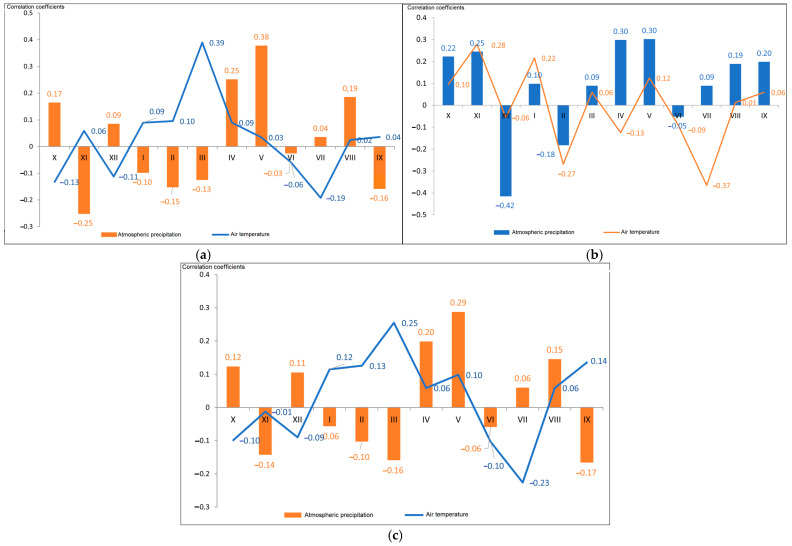
Correlation coefficients (CCs) of the oak radial growth indices with average air temperature and total precipitation based on the Voronezh Meteorological Station data for the months from October to September: (**a**) plus trees, (**b**) family 1, (**c**) family 2.

To analyze the relationship between the TRW and monthly air temperature and precipitation, the Pearson’s CCs were calculated between all chronologies, between the TRW and temperature and precipitation for a period of 12 months, from October of the previous year to September of the following year (Figure 8).

Correlation analysis for plus trees showed that, in general, the tree-ring chronologies correlated with the temperature of the same growing season (April–July) and differed only in intensity and significance of the monthly CC, which is typical for many chronologies of the East European Plain [81,82,83,84]. As the climate continentality increases, trees become more sensitive to moisture and vegetation temperature of both the same year and the previous year (Figure 8).

Precipitation in the autumn–winter period and weather conditions at the onset of active vegetation in April and May were of great importance for the radial growth of oak. Regarding precipitation in the period preceding the vegetation, the strongest correlation of precipitation with growth was found for plus trees in October of the previous year and April–May of the following year. The air temperature had a large limiting effect on oak growth, especially in March (*r* = 0.39, *p* < 0.05). The oak tree-ring indices showed a rather moderate correlation with March temperatures, but statistically significant at a confidence level of 0.95, and a slightly lower correlation with February and April temperatures, especially for plus trees and family 2. The direct effect of temperature on radial wood growth was often observed at the onset of the season, when unusually cold weather caused a delay in growth activation after winter dormancy.

The strongest positive relationship of growth was observed with precipitation in May and April for all studied families. For family 1 and plus trees, weaker correlation of growth was observed with precipitation in March and June. For these trees, a significant negative correlation of growth was found also with the average monthly temperatures in April, May, June and August.

A strong correlation of growth was found with precipitation in December of the previous year for all families.

The average monthly winter temperatures were lower in the years of high growth than in the years of low growth. This may indicate the negative impact of thaws on winter hardiness and growth of oak. High temperatures in May had a negative impact on growth, as they increased dry conditions during that month. It is obvious that temperature and precipitation are the main factors influencing the annual growth of pedunculate oak, and they are closely interrelated.

Precipitation in May had the greatest significant positive effect on growth of family 2 in the same year (*r* = 0.29, *p* < 0.05). Negative correlations between growth and precipitation were typical for March and December.

**Figure 9:**
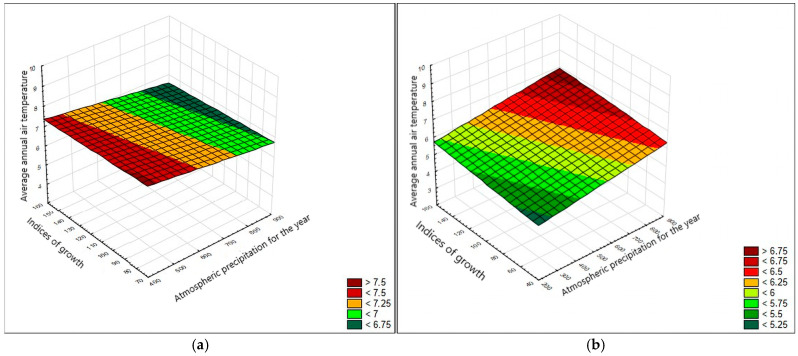
Spline of oak-growth indices, air temperature and precipitation for the entire period of the study by tree ring chronology: (**a**) family 1, (**b**) plus trees.

Correlation analysis demonstrated that precipitation was also a limiting factor for the radial growth; the correlation of growth with temperature proved to be slightly higher (*r* = 0.54, *p* < 0.05) than with precipitation (*r* = 0.42, *p* < 0.05) for the entire period of the study by tree ring chronology. The correlations were positive and significant (Figure 9).

## 3. Discussion

### 3.1. Radial Growth Responses to Drought

The TRW can fully reflect the relationship between the radial growth of trees in the study area and climatic factors. Statistical characteristics (high standard deviation, MS, signal-to-noise ratio and pronounced population signal of collected samples) indicate that the investigated chronologies contain information about a climate response [30].

Our research confirmed these assumptions and found that at the border of the habitat range, in the forest-steppe zone of the Central Russian Upland, pedunculate oak plus trees and their seed progeny carry a climate response, which is similar to oak studies in other regions [85,86,87,88,89]. However, the degree of populations’ specific response to climatic factors may differ and depend both on species characteristics [90] and on the ecological/geographical conditions of their habitat [91,92]. Besides differences in climate response, those in adaptive responses to drought exist [90]. A decrease in the intensity of climate response among the progeny of plus trees can be explained by both the age (younger trees in families 1–4 as compared with the mature plus trees) and the genetic heterogeneity of the progeny, which often do not inherit the entire set of plus-tree traits. In general, plus trees are more sensitive to climate and drought, which is consistent with [86,93]. With age, the response to abnormal temperatures in plus trees increases.

The high-frequency climatic cycles reflected in radial growth were determined by variation in the intensity of tree growth, the stage of ontogenesis, or short-wave climate fluctuations across years [94].

Within the chosen time-frame periods of drought, the course of tree-ring chronologies was not the same; the reactions of individual plus trees and families have been recorded in terms of the time of response to drought and its duration. Oak growth decreased under the influence of medium- and long-term droughts caused by low precipitation, as well as high maximum temperatures of the warm period, which can be associated with an increase in the rate of evapotranspiration in the area [95]. Thus, growth indices were used to identify differences in radial growth before and after years with intense drought between the studied families and individual plus trees. Comparison of the sequences of narrow and wide rings allowed synchronization of the compared time series across families and individual plus trees. Periods in which the majority of trees form narrow or wide rings were designated as pointer years. It was assumed that such years are characterized primarily by various weather anomalies [96], such as droughts in our study.

In the population of pedunculate oak, we can single out dendrophenotypes that differ one from another in their reactions in the periods before, during and after drought and in sensitivity to climatic factors, which is confirmed by the direction and strength of correlations of tree-ring chronologies with climatic factors. The phenotypic and genotypic variation of individuals is an adaptive response to the effects of climatic factors, in particular, drought, and can be the basis for the selection of genotypes both among agricultural crops and trees [97,98].

Thus, we can assume the presence of genotypic variability among individual plus trees and their progeny, which determines differences in dendrophenotypic reactions.

### 3.2. Climatic Factors Limiting Radial Growth

In general, the possibility of counteracting the dry periods of the summer months should be considered for plus trees of pedunculate oak in combination with the temperatures of the autumn–winter period due to the high degree of their influence. Some investigators noted a negative correlation of radial growth with January temperature, explaining this by the acceleration of physiological processes in trees in warm winters and a greater consumption of nutrients than in cold winters, which affects wood growth during the growing season. A positive relationship between the growth of early oak wood and air temperatures in December of the previous year and March of the same year is known. Analysis of data obtained in [99] revealed a number of factors that limit oak growth; the high temperature of early summer stands out among them. In the forest-steppe, the main factor influencing oak growth is air temperature; herewith, severe droughts (1972 and 2010) were observed both in the year of drought and 2–3 years after, which is associated with precipitation deficit and high air temperatures. It is a known fact that oak has a more noticeable slow-action effect, i.e., unfavorable conditions in a given summer can affect the TRW in the next year or subsequent years. In temperate northern Spain, the radial growth of old-growth pedunculate oaks was limited by cold winter and summer temperatures, as well as dry summer conditions [87,88]. In Spain, warmer spring conditions promoted long-term oak growth in cold and wet sites but contributed to its decline in warm and dry sites [89]. Our results show that the lack of precipitation in spring and high temperatures in summer reduce oak growth.

The growth is believed to be closely related in many trees to the growth in the previous year [100]. This relation indicates that fluctuations in growth can also occur under the influence of internal factors. The current environmental conditions have only a modifying effect, and significant disturbances in the tree’s own rhythm of growth occur only in the years with weather anomalies. It is important to conclude that neither air temperature nor precipitation has a decisive effect on oak growth separately, but in combination, these two climatic factors play a leading role in the growth process. For pedunculate oak, the limiting factor is still air temperature, but the role of precipitation is also significant and positive, especially during the warm period (May–August).

On average, there was a decrease in growth of plus trees and their progeny both in the year of drought and 2–3 years after, which was associated with a lack of precipitation and high air temperatures. The individual adaptive potential of trees will be manifested in the speed of recovery from growth depression, and can depend on the tree’s condition, age, origin and habitat. Data obtained in [6,86,93] demonstrated that weakened trees had a more pronounced climate response and were more sensitive to drought.

The analysis of the anatomical structure of wood in [85] has shown the possibility of changing the type of wood in *Q. ithaburensis*, *Q. boissieri* and *Q. calliprinos* in dry years from ring-porous to semi-ring-porous. No similar changes were found for pedunculate oak, which may indicate different anatomical and physiological adaptation of pedunculate oak trees in response to drought.

To manage vulnerable forest plantations, it is necessary to formulate their spatial and dendrophenotypic characteristics, which will make it possible to further remove individuals with weak adaptive potential and to reallocate resources via assisted migration [101]. Species inhabiting a certain region are adapted to changes in climatic conditions of the past millennia [102]. However, species less adapted to the new, drier conditions may disappear from many regions in the coming decades [103,104]. Therefore, understanding how species with different traits and strategies respond to drought is crucial for predicting future scenarios in this region and for making management decisions [87].

Thus, our study revealed a high variability of dendrophenotypic responses and determined systems of significant climatic factors affecting the growth of pedunculate oak on the whole and for plus trees in particular, which is important for predicting the state of forest plantations under climate change conditions.

There are differences in the response of radial growth to climate parameters of pedunculate oak between plus trees and their half-sib progeny. The dendrophenotypic reactions of plus trees and their half-sib progeny of four plus trees before, during and after drought were characterized for this species for the first time.

Thus, the external climatic factors impact the individual adaptive potential and growth features of trees. The radial growth is, thus, an individual biological characteristic carrying the pronounced climate response.

## 4. Materials and Methods

### 4.1. Studied Area and Trees

**Figure 10:**
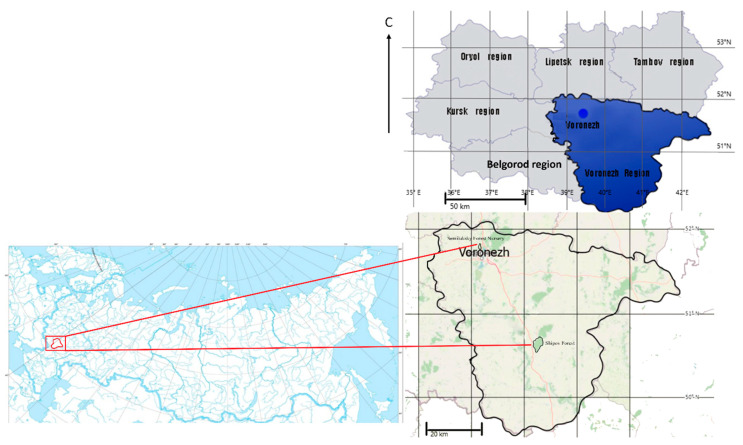
Study area: Shipov Forest, Voronezh Region, Central Federal District, Russia.

The research was carried out in the natural oak population of Shipov Forest of the Voronezh Region (50°46′00″ N, 40°20′00″ E) (20 plus trees), and in the Semiluksky Forest Nursery (51°69′42′′ N, 39°00′17′′ E) (half-sib progeny of four plus trees) (Figure 10).

In September 2022, to identify the climate response and dendrophenotypic features, wood core samples were collected from 97 pedunculate oak trees, including 20 plus trees in Shipov Forest (142 years old on average) and seed progeny from half-sib families in the Semiluksky Forest Nursery for four of them (Table 5). The length of the age series at a height of 1 m from the ground level in the progeny was 40 years.

The examples of a plus tree and wood core samples are presented in Figures S1–S5 in the Supplementary Materials. The soil of the studied sites was medium-thick clayey chernozem on loess-like carbonate clay of average mechanical composition. It was slightly acidic with thickness of the humus horizon 50–75 cm and humus content in the arable layer 5.1–5.9%.

### 4.2. Climate of the Study Area

The Shipov Forest is located at the border of the forest-steppe and steppe zones, so precipitation is of greater importance for growth there.

To identify unfavorable periods for pedunculate oak based on climatic factors, a detailed analysis of the following parameters was carried out: the amount of precipitation and average annual air temperature for the period from 1873 to 2022. Periods of abnormally significant droughts and their response reflected in the radial growth of pedunculate oak were noted and selected for further analysis. Two time periods of 1991–2020 and 1987–2016 were used in our analysis of climate data because the 1991–2020 period represents a 30-year period—a climate norm according to the World Meteorological Organization (WMO) data and 1987–2016 is the most recent 30-year-long period of greatest climate warming.

### 4.3. Wood Core Measurements

Oak wood core samples were extracted with a Pressler age drill at a height of 1.3 m. The cores were taken along a randomly oriented radius. After extraction, the cores were wrapped in paper and transported in a solid container to prevent breakage. Before measurements, the cores were soaked in water, acquiring a brighter color. The cores were cleaned with a sharp blade in the direction from the bark to the center and then framed for measurement and dating.

The LINTAB-6 measuring device with the TSAP–Win software was used to date and measure the tree-ring width (TRW).

The average values of the radial growth of oak for each calendar year were determined by summing up the TRW of all available samples for a particular year and dividing it by the number of samples.

The mean sensitivity (MS) coefficient was calculated as the average absolute difference between consecutive ring widths, normalized by the average ring width. It is a metric that quantifies the year-to-year variability in tree ring widths, providing insights into environmental fluctuations and stress factors affecting tree growth. High MS indicates significant annual variation in growth, often reflecting a climate with fluctuating conditions, such as irregular precipitation or temperature. Conversely, low MS suggests more stable growth conditions with less environmental stress. This coefficient is particularly valuable for understanding a tree’s or population’s responsiveness to climate, especially in regions where climate variations strongly influence growth. Trees in continental climates with marked seasonal changes, for example, often have higher MS due to pronounced shifts between warm and cold seasons.

To rule out the effect of age and to adequately compare radial growth with fluctuations in climatic characteristics, we calculated the relative indices of radial growth of forest stands in the TREND program [105].

Data standardization involved indexing the data from the TRW measurements by the generally accepted formula: $I=\frac{i_{f}}{i_{s}}\times 100\%$, where *I* is a relative index (%), *i_f_*—the actual width of annual tree ring, *i_s_*—the smoothed values of the growth rate, depending on age, calculated in the TREND program [105] using the sliding and polynomial smoothings.

The average coefficient of correlation (*r*_av_) between standardized series of radial tree growth indices was calculated to assess the common signal strength between tree-ring series. This coefficient is calculated by first standardizing each tree’s radial growth series, which typically involves removing age-related growth trends to isolate climatic or environmental signals by dividing each ring width by a fitted growth trend line (e.g., a spline or linear detrending method). This removes long-term growth trends, focusing on high-frequency climate signals. The standardized indices are then cross-correlated for each pair of tree-ring series in the dataset using Pearson’s correlation coefficient, reflecting the extent to which the two trees exhibit synchronous growth patterns over time. The mean of these pairwise correlation coefficients is then taken to produce the average value correlation coefficients (CCs). This single value indicates the degree of shared growth variability across the entire dataset, with higher values indicating stronger common signals due to climate or environmental influences. This metric is useful in dendrochronology to assess the coherence of the climate signal within a sample site, as trees that experience similar environmental conditions (e.g., precipitation, temperature) tend to have high inter-series correlation values. High average inter-series correlation suggests that the environmental factors driving growth are consistent across the sampled trees.

The expressed population signal (EPS) metric was calculated to determine how well a sample of tree-ring series represents the hypothetical “true” population signal, reflecting common climatic or environmental factors. EPS is used to assess whether the sample size of trees is sufficient to capture the regional environmental signal reliably. It was calculated based on *r*_av_ and number of trees in the sample used in the analysis (n) as follows: EPS = $\frac{n\times r_{av}}{1+(n-1)\times r_{av}}$. Larger sample sizes help stabilize the common signal and reduce the impact of individual tree noise. EPS ≥ 0.85 is generally considered to indicate an acceptable level of representativeness for dendrochronological studies, meaning the sample reliably reflects the common signal. EPS < 0.85 suggests that the sample may not adequately represent the true population signal, often implying that a larger sample size or more uniform growth response is needed. EPS is especially useful when comparing different sites or periods, as it helps determine the confidence in the climate signals derived from the tree-ring dataset.

The signal-to-noise ratio (SNR) was calculated to measure the strength of the common environmental or climatic signal relative to the individual, random variations (noise) among tree-ring series in a sample. A higher SNR indicates that the sample’s common signal (often driven by climate) is stronger and more reliable, relative to random, non-climatic variability. It is calculated based on *r*_av_ and the number of series (trees) n as follows: SNR = $\frac{n\times r_{av}}{1{-r}_{av}}$. A high SNR means that the collective tree-ring series accurately reflect the common environmental signal. SNR is closely related to the EPS, but SNR directly quantifies the ratio, whereas EPS provides a threshold. SNR is used in dendrochronology to evaluate the robustness of tree-ring datasets, ensuring that the data provide a reliable climate signal, which is particularly valuable in reconstructing past climates.

It should be noted that precipitation less than 50% of the norm is considered as an indicator of very severe drought, and 50–70% as severe drought [106]. In the period from 1882 to 2022, severe droughts were registered in 1936, 1939, 1972, 1992, 2010 and 2011.

The main statistical characteristics of the dendrochronological series (average values, coefficient of variation, standard deviation, probable error, research accuracy, etc.) were calculated in STATISTICA 13.0 [107].

### 4.4. Statistical Analysis

Using the TSAP–Win Professional software v.4.67 [108], the similarity coefficients of the TRW chronologies for each sample with the average chronology, the synchronicity coefficient (GLK), the synchronicity level (GSL), the correlation coefficient (CC) between the TRW chronologies of each sample and the average chronology, and the cross-dating index (CDI) were calculated for the studied tree samples [77].

The GLK (%) estimates the number of unidirectional changes from year to year between two chronologies [77]: GLK = $\frac{n^{+}}{n-1\;}\times 100\%$, where *n*^+^ is the number of year segments of two chronologies that coincide in direction, and *n* is the duration of the time interval of the compared chronologies.

The GSL ranks the level of synchronicity: “0” for GLK ≤ 56, “1” for GLK = 57–60%, “2” for GLK = 61–67%, “3” for GLK ≥ 68% in the generated individual tree-ring chronologies [77].

The cross-dating index (CDI) is a combination of the Student’s *t*-test, CC and GLK. It is expressed in % and calculated in TSAP–Win Professional [108]. The CDI values of more than 10% are accepted to be reliable.

To reveal the extremes of the relative growth indices corresponding to the extremes of climatic factors (total annual precipitation, average air temperature) that limit the growth of pedunculate oak and to examine the frequency characteristics of tree-ring data and identify periodic or cyclical patterns, we carried out a spectral and cross-spectral analysis with smoothing using the Hamming weights in the STATISTICA 13.0 program [107]. These methods can help understand how climatic or environmental signals, such as drought or temperature cycles, influence tree growth at various frequencies. Spectral analysis decomposes tree-ring series into its frequency components, allowing to see which frequencies (or periodicities) are most prominent in the data. This analysis might reveal cycles in growth that correspond to known climate cycles. The tree-ring series is first transformed using a Fourier transform to identify the power or amplitude of each frequency component. This reveals dominant cycles in growth patterns, indicating recurring climate conditions.

Cross-spectral analysis is an extension of spectral analysis that assesses the relationship between two time series (e.g., tree-ring series from two different locations or tree-ring data and climate records) in the frequency domain. It allows to determine whether two series share similar cycles and how they may be phase-shifted relative to each other. First, both series are transformed to the frequency domain, and then the cross-spectrum is calculated to measure the shared power at each frequency. This shows how closely the signals are aligned across different frequencies, which can indicate shared environmental influences on tree growth. Smoothing with Hamming weights is applied to reduce noise in the frequency data and clarify meaningful patterns. Hamming weights, a type of window function, are used for this purpose. The Hamming window assigns different weights to data points, with higher weights near the center, which helps minimize abrupt changes and spectral leakage. The Hamming window is applied by multiplying each data point in the spectral series by the corresponding weight. This smooths out high-frequency noise, making significant cycles easier to detect. By reducing high-frequency noise, smoothing with Hamming weights highlights key periodic signals, making it easier to interpret the dominant frequencies and shared patterns between series.

These techniques help clarify how climate oscillations (like drought cycles) influence tree growth at specific periods. Spectral and cross-spectral analyses are especially useful for investigating climate patterns over multiple time scales and for comparing regional similarities in climate influences on tree growth.

To assess the influence of climate on the tree growth, monthly series of temperature and precipitation obtained at the Voronezh Meteorological Station (51°42′55″ N, 39°12′57″ E) were used.

To assess the quantitative relationship between the investigated relative oak growth indices and climatic factors (precipitation and average air temperature), the Pearson linear correlations (CCs) were calculated using Microsoft Excel 2020. According to Chaddock’s scale, the relationship is considered weak from 0 to 30%, moderate from 31 to 50%, significant from 51 to 70%, high (close) from 71 to 90%, and very high (very close) from 91% to 100%.

To characterize the families and individual trees, we used the indices of dendrophenotypes proposed in [40], such as indices of resistance *Rt* = Gd/Gprev), resilience *Rs* = Gpost/Gprev, and recovery *Rc* = Gpost/Gd), where Gprev is the average growth (measured using either TRW or BAI) over two–three years before a stress (drought), Gd—the average growth during the stress, and Gpost—the average growth within two to three years after the stress.

The effect-size index of the meteorological factors on the radial growth of wood was calculated as the ratio of the factorial sum of squares (*Df*) to the total sum of squares (*Dc*) of the dispersion complex (STATISTICA 13.0) as follows: $\eta^{2}=\frac{Df}{Dc}$.

In this case, the values of the relative growth indices were used as the resulting dependent traits or dendrophenotypes, and the meteorological factors as independent variables. If the actual value of the *F*-criterion was higher than the tabulated *Ff* > *Fst*, the statistical significance of the effect-size index of the meteorological factors on the radial growth of wood as a whole was recognized.

## 5. Conclusions

Dendrochronological analysis of the TRW and climatic factors enabled us to identify periods of the past that correspond to severe droughts and made it possible to evaluate the characteristics of the climate response of plus trees and their half-sib progeny, and also to reveal the intrapopulation adaptive potential of plus trees in relation to drought.

It was shown that:

1. The identified years of the most severe droughts (1939, 1972 and 2010) were characterized by a lack of precipitation for at least two previous years and extremely high average and maximum air temperatures of the warm period (May–August).
2. The high frequency of abnormal rings in 142-year-old plus trees was observed during the drought years and for the following next three years with some variations.
3. Air temperature was the main limiting factor for the pedunculate oak annual growth. However, precipitation patterns also made a significant contribution to the growth, especially in recent decades.
4. Cross-spectral analysis of the total TRW and atmospheric precipitation showed the most significant cycles in the growth in the following order: 12, 6, 9, 4 and 18 years. Plus trees featured a pronounced Brückner–Egeson–Lockyer cycle (32 years). When analyzing the cross-spectral density of air temperature series and growth indices for total oak wood, 2–3-year cycles were the most pronounced; the prevalence of an 18-year cycle was also revealed.
5. Reduction in the tree radial growth due to drought stress varied significantly between families, indicating differences in physiological and ecological regulation strategies. The resilience of plus oak trees to drought was higher than that of the progeny. Drought was found to result in reduced growth during the year(s) following the drought; hence, the minimum growth occurred after the actual climate event.
6. Plus trees had a high adaptive potential due to the resistance and resilience of radial growth to drought (Rt = 1.29, Rs = 1.09). The offspring of families 1 (Rt = 0.89, Rs = 0.89) and 2 (Rt = 1.04, Rs = 0.87) had similar resistance and resilience. They were lower for the offspring of families 3 and 4.
7. Precipitation in the autumn–winter period and weather conditions at the onset of active vegetation in April and May were of great importance. The influence of air temperature in March on oak growth is large (*r* = 0.39, *p* < 0.05). The strongest positive relation between precipitation and growth was found in May of the same year, up to 0.38.

Plus trees were a special object of our study due to their exceptional phenotype and health at the age of over 100 years. These trees are considered as prospective etalon trees from the economic point of view. We studied and presented their dendrophenotypes with respect to their general climatic response and its variation in their progeny. We also compared the dendrochronological response of plus trees with the available data for oak in general. Comparison with the results of other studies showed a pronounced climatic response in oak [30,85,86,87,88,89]. Different oak responses to climatic factors are discussed considering their place of growth, population and species specifics, which can significantly determine the influence of leading climatic factors, as well as the rate and dynamics of the annual ring growth of wood. Differences were found between plus trees and their progeny, which we discussed considering the age and genetic heterogeneity of the progeny [86,90,93]. Based on the obtained data, we assume the presence of high genotypic variation reflected in the differences in dendrophenotypic reactions between individual plus trees and their progeny. In continuation of our study, involving ordinary (non-plus) trees and an additional larger number of old-age plus trees in comparison, we plan to describe in more detail the observed biological features of the plus trees.

## Figures and Tables

**Table 1:** Total precipitation and average temperature in July in drought years in the Voronezh region.

Drought Year	Total Precipitation in July, mm	Average Temperature in July, °C
1938	10	24.7 ± 0.003
1939	15	22.3 ± 0.003
1971	31	20.6 ± 0.002
1972	37	23.5 ± 0.003
1992	50	18.9 ± 0.001
2010	38	26.4 ± 0.002

**Table 2:** Statistical indicators of oak radial-growth chronologies for four families (1–4) and 30 plus trees.

Indicator	1	2	3	4	Plus Trees
Time span of chronologies	1982–2022	1983–2022	1983–2022	1982–2022	1881–2022
Number of wood core samples	15	22	15	15	30
MS	0.26	0.25	0.31	0.25	0.29
*r*_av_	0.429	0507	0.517	0.564	0.547
EPS > 0.85 *	1985	1987	1984	1983	1883

Note: MS—mean sensitivity of chronology coefficient, *r*_av_—average coefficient of correlation between standardized series of radial tree growth indices measured in the tree sample for a given time period, * the first year in chronology, starting from which expressed population signal (EPS) > 0.85.

**Table 3:** Values of synchronicity coefficients (GLK), synchronicity level (GSL), correlation coefficients (CC) and cross-dating indices (CDI) of individual chronologies of radial growth of oak trees for total annual radial width (ARW), early wood width (EWW) and late wood width (LWW).

Individual Chronologies of Radial Growth	GLK, %	GSL	CC, %	CDI, %
Family 1				
ARW	66 ± 1.5	2	79 ± 1.5	31 ± 1.1
EWW	65 ± 1.8	2	66 ± 2.3	21 ±1.2
LWW	73 ± 2.5	3	78 ± 1.3	32 ± 0.8
Family 2				
ARW	78 ± 2.6	3	79 ± 2.6	76 ± 2.5
EWW	76 ± 2.3	3	79 ± 2.5	55 ± 1.5
LWW	74 ± 2.4	3	66 ± 2.4	26 ± 0.2
Family 3				
ARW	60 ± 1.3	1	65 ± 1.5	20 ± 0.3
EWW	55 ± 0.8	0	64 ± 1.4	18 ± 0.2
LWW	52 ± 1.2	0	70 ± 1.9	35 ± 0.4
Family 4				
ARW	62 ± 1.5	2	58 ± 2.3	36 ± 0.2
EWW	50 ± 1.3	0	61 ± 2.5	15 ± 0.1
LWW	65 ± 1.8	2	59 ± 1.6	16 ± 0.1
Plus trees				
ARW	81 ± 1.9	3	79 ± 1.4	78 ± 1.2
EWW	80 ± 2.3	3	82 ± 1.6	75 ± 0.9
LWW	82 ± 2.4	3	85 ± 1.8	80 ± 2.3

**Table 4:** Average resistance (*Rt*), resilience (*Rs*) and recovery (*Rc*) indexes in four families (1–4) and plus trees in response to drought stress (2010–2012 years).

Trees	*Rt*	*Rs*	*Rc*
Family 1	0.89 ± 0.002	0.89 ± 0.002	0.99 ± 0.002
Family 2	1.04 ± 0.003	0.87 ± 0.001	0.84 ± 0.001
Family 3	0.75 ± 0.001	0.68 ± 0.001	0.82 ± 0.001
Family 4	0.71 ± 0.001	0.63 ± 0.001	0.62 ± 0.001
Plus trees	1.29 ± 0.001	1.09 ± 0.004	0.85 ± 0.004

**Table 5:** Mean silvicultural characteristics with standard error (s.e.) of the studied 97 pedunculate oak trees including 20 plus trees and families of four of them.

Oak Trees	Number	Age ± s.e., Year	Height ± s.e., m	DBH ± s.e., cm
Plus trees	20	142 ± 3.0	30 ± 0.8	49 ± 1.6
Family 1	20	40 ± 1.0	19 ± 0.09	23 ± 0.5
Family 2	20	40 ± 1.0	20 ± 0.6	27 ± 0.8
Family 3	20	40 ± 1.0	21 ± 0.2	28 ± 0.9
Family 4	17	40 ± 1.0	22 ± 0.6	29 ± 0.9

## Data Availability

The original contributions presented in the study are included in the article, further inquiries can be directed to the corresponding author.

## Supplementary Materials

The following supporting information can be downloaded at: https://www.mdpi.com/article/10.3390/plants13223213/s1, Figure S1: An example of a plus tree; Figure S2: A fragment of a wood core sample from a tree in Family 1 originated from the eastern part of the Central climatic region, northern forest-steppe subzone, Russia; Figure S3: A fragment of a wood core sample from a tree in Family 2 originated from the Central climatic region, southeastern forest-steppe (Voronezh Region, Russia, “local” climatype); Figure S4; A fragment of a wood core sample from a tree in Family 3 originated from the Southern climatic region, steppe zone (Republic of Dagestan, Russia); Figure S5: A fragment of a wood core sample from a tree in Family 4 originated from the Northwestern climatic region, mixed forest zone (Novgorod Region, Russia).
