# Supplementary material for: Climate Response and Radial Growth Dynamics of Pedunculate Oak (Quercus robur L.) Plus Trees and Their Half-Sib Progeny in Periods of Severe Droughts in the Forest-Steppe Zone of Eastern Europe
Source: Plants (Basel). 2024 Nov 15;13(22):3213. doi: 10.3390/plants13223213 (PMC11598455; doi:10.3390/plants13223213)
Supplement: Supplementary file 1 [file plants-13-03213-s001.zip › plants-3281200-supplementary.pdf]

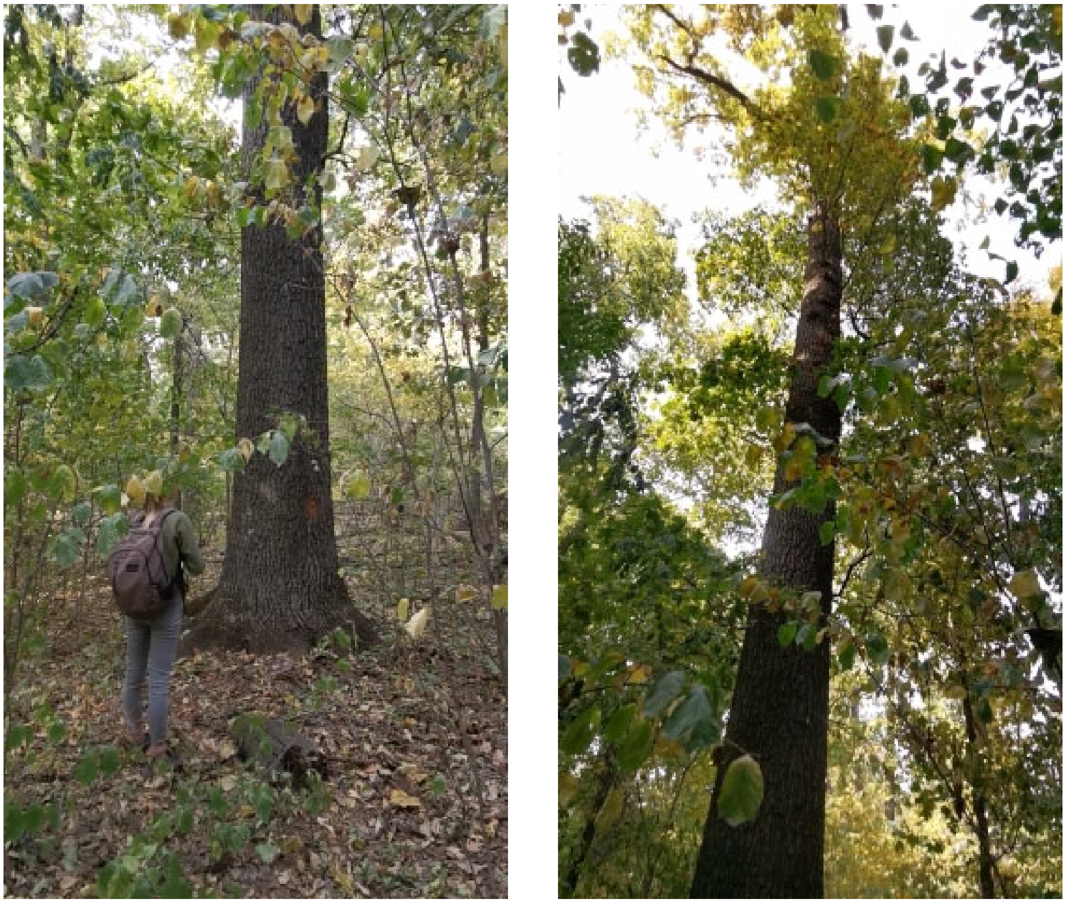

Figure S1. An example of a plus tree.

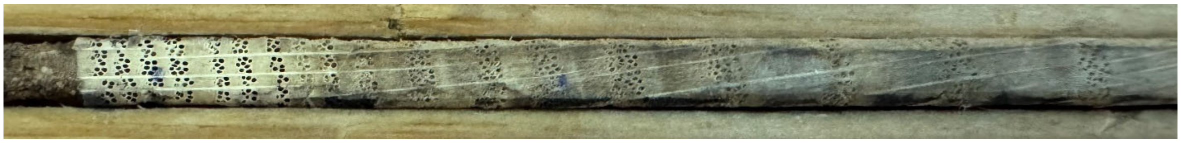

Figure S2. A fragment of a wood core sample from a tree in Family 1 originated from the eastern part of the Central climatic region, northern forest-steppe subzone, Russia.

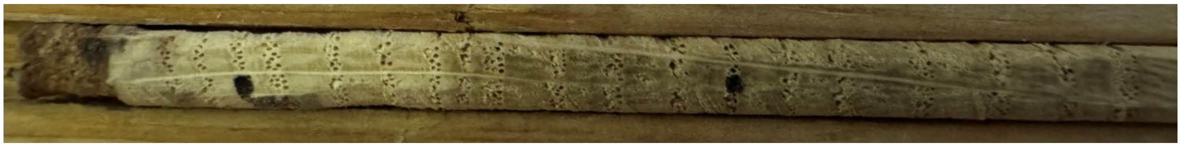

Figure S3. A fragment of a wood core sample from a tree in Family 2 originated from the Central climatic region, southeastern forest-steppe (Voronezh Region, Russia, “local” climatype).

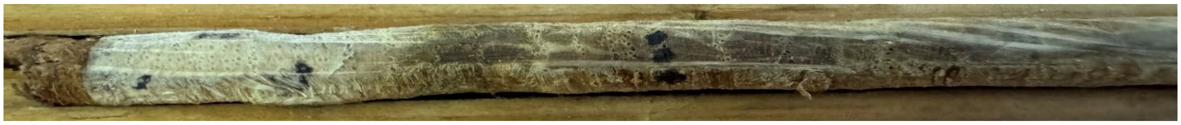

Figure S4. A fragment of a wood core sample from a tree in Family 3 originated from the Southern climatic region, steppe zone (Republic of Dagestan, Russia).

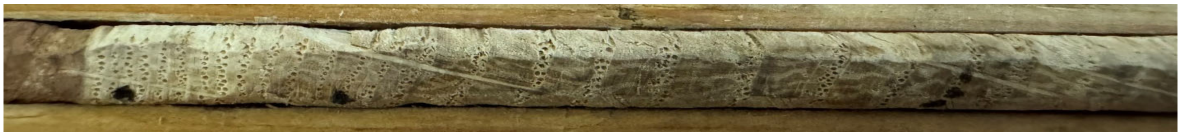

Figure S5. A fragment of a wood core sample from a tree in Family 4 originated from the Northwestern climatic region, mixed forest zone (Novgorod Region, Russia).
